# Waveform-Feature-Driven Diagnosis and Physics-Constrained Self-Correction of Representative EMT Component Implementation Faults: A Multi-Agent Feasibility Study

**DOI:** 10.3390/s26185789

**Published:** 2026-09-12

**Authors:** Jieran Zhang, Jie Zhang, Pan Wu, Jin Xu, Keyou Wang

**Affiliations:** 1 School of Electrical Engineering, Shanghai Jiao Tong University, Shanghai 200240, China; panghuwu@sjtu.edu.cn (P.W.); xujin20506@sjtu.edu.cn (J.X.); wangkeyou@sjtu.edu.cn (K.W.); 2 State Key Laboratory of HVDC, Electric Power Research Institute, China Southern Power Grid, Guangzhou 510663, China; zhangjie5@csg.cn; 3 National Energy Power Grid Technology R&D Centre, Guangzhou 510663, China; 4 Guangdong Provincial Key Laboratory of Intelligent Operation and Control for New Energy Power System, Guangzhou 510663, China

**Keywords:** fault diagnosis, waveform feature extraction, decision support system, electromagnetic transient simulation, Feature Selective Validation, multi-agent system, large language model, power system reliability

## Abstract

Latent faults in electromagnetic transient (EMT) components can alter initialization, switching-event, and history-state semantics while producing sparse or small waveform discrepancies. This study formulates a reference-model-based verification as a closed-set, waveform-guided diagnosis and constrained source-repair problem. Global, local-window, and event-level evidence rank fault mechanisms or trigger abstention. Repairs must pass compile-feasibility, behavioral-conformance, and applicability-aware physics-and-discretization audit gates. A multi-agent workflow implements this process. InvSqrt and single-phase-breaker faults were corrected to exported-waveform precision; the breaker record contained only two nonzero differences among 20,000 samples. The VARRL correction yielded 0.1% residual node-voltage RMS differences. A within-case VARRL extension across four matched conditions and three source variants exposed condition-dependent activation, a weakly separated effect of 0.018% RMS relative difference, a smaller inseparable effect routed to expert review, and a mixed-snapshot variant that reduced reference error but violated a discretization contract. An author-constructed ten-candidate challenge set spanning three component structures compiled in RSCAD FX 2.3 CBuilder; the deterministic audit returned Pass for three designed semantics-preserving refactorings, Reject for six isolated rule violations, and Expert review for one underspecified temporal convention. These results provide mechanism-level and preliminary audit-coverage evidence rather than population-level fault frequency, independent classification accuracy, general rule sufficiency, or formal verification.

## 1. Introduction

Electromagnetic transient (EMT) simulation underpins protection validation, converter-interaction assessment, and hardware-in-the-loop testing in modern power systems [1,2]. Recent studies have also reported the application of small- and medium-capacity VSC-HVDC systems to electromagnetic loop-network problems in power systems [3], further highlighting the need for credible EMT-oriented modeling and validation. Model fidelity is therefore critical: latent implementation faults, including initialization errors, switching-event inconsistencies, and history-state update errors, are often observable only through waveform signatures, yet they can propagate into protection-setting verification, HIL assessment, and reliability analysis.

Terminal waveforms are often the principal observable evidence for assessing an EMT component whose reference implementation is opaque. Global bias, local distortion, and event anomalies can indicate initialization, switching-logic, or history-state faults, but the same waveform mismatch may also arise from parameter settings or discretization choices. Custom-component verification therefore requires more than a scalar error: the observed symptom must be connected to a plausible source mechanism and an admissible correction, following a signal acquisition–feature extraction–fault reasoning–decision support chain that links EMT model validation with waveform-based diagnosis of electrical signals.

This difficulty is consistent with recent industry guidance and project experience on EMT model verification. NERC guidance emphasizes that high-quality, facility-specific EMT models require documented model-quality and verification practices, and explicitly notes that using a real-code EMT model does not by itself establish validation for the intended equipment [4,5]. A recent NERC industry survey reports heterogeneous EMT model-quality practices, incomplete formal processes in part of the surveyed organizations, and continued dependence on external expertise [6]. Experience reported by a transmission system operator further shows that manufacturer-supplied EMT models are commonly delivered as black-box implementations, that discrepancies and inconsistencies may be found during model testing, and that verification requires substantial expert effort [7]. These sources establish the practical importance and resource burden of EMT model verification, but they do not provide a population-level occurrence rate for the source-level mechanisms studied here. Accordingly, this paper reports project-derived, mechanism-level feasibility evidence rather than an industry-wide fault-frequency claim.

Existing methods cover only parts of this task. Parameter-identification methods calibrate continuous parameters while presuming a valid implementation structure [8,9,10]. Automated program repair and recent LLM agents iteratively edit code under compiler and software-test feedback [11,12,13,14], but compilable EMT code can still violate state continuity, event logic, or discretization consistency. Generic code models and power-system LLM applications also require domain-specific safeguards [15,16,17,18,19].

Physics-informed learning embeds governing knowledge during training [20]; here, physical knowledge instead screens discrete source edits. FSV-related methods quantify amplitude and structural waveform differences [21,22,23], but do not map those differences to source mechanisms. The unresolved gap is therefore a closed loop that combines waveform evidence, mechanism diagnosis, source repair, and physics-and-discretization admissibility. To the best of the authors’ knowledge, closed-loop source-level correction of EMT component implementations under combined waveform-feature and physics-audit feedback has not been reported.

This paper addresses the above gap by studying reference-model-based verification of inspectable EMT component implementations. The specific research object is waveform-guided diagnosis and constrained source repair of implementation-induced behavioral non-conformance. Given a trusted black-box reference waveform, an inspectable candidate white-box implementation, and its exported waveform, the task is to combine global, local-window, and event-level waveform evidence with candidate-source inspection to select the most plausible source-level fault mechanism from a predefined library. The mechanism library covers initialization, event/history-state, and coupled history-update faults, with explicit no-actionable-fault and expert-review outcomes when the available evidence is insufficient. A candidate repair must pass a compile-feasibility gate and must then satisfy both behavioral-conformance conditions and applicable physics-and-discretization admissibility checks. The multi-agent workflow is used as the implementation and traceability architecture of this process rather than as the primary scientific object of study [24].

The evaluation retains three project-derived cases: InvSqrt, the single-phase breaker, and VARRL. The VARRL case is extended across four matched conditions and three executable source variants to examine condition-dependent activation. A separate author-constructed challenge set uses ten CBuilder-compilable candidates spanning algebraic, event-driven, and history-state structures to exercise all six audit-rule categories. The controlled variants are mechanism probes, not additional project-derived cases.

The contributions are threefold. First, we formulate reference-model-based verification of EMT component implementations as a closed-set behavioral non-conformance diagnosis problem under reference-side opacity, with explicit no-actionable-fault and expert-review outcomes. Second, we combine global, local-window, and event-level waveform evidence to characterize temporally sparse implementation faults that can be strongly diluted by full-record metrics. Third, we formulate source repair as a triple-gate problem requiring compile feasibility, behavioral-conformance improvement, and applicability-aware physics-and-discretization admissibility. The three project-derived cases provide mechanism-level feasibility evidence for the predefined fault classes. The within-case VARRL extension additionally shows that lower waveform error does not imply an admissible implementation; the challenge set evaluates designed refactorings, isolated violations of R1–R6, and an underspecified temporal convention.

The remainder of this paper is organized as follows. Section 2 presents the problem formulation and multi-agent architecture. Section 3 introduces the workflow, objective, and evaluation metrics. Section 4 describes the experimental setup. Section 5 reports three project-derived benchmark demonstrations, the within-case VARRL source-variant extension, a cross-component offline audit challenge set, and the offline sensitivity analysis. Section 6 and Section 7 discuss the findings and conclude.

## 2. Problem Formulation and Multi-Agent Architecture

### 2.1. Problem Formulation

The task is formulated as reference-model-based implementation verification rather than the reconstruction of the proprietary reference model. Let $u_{j}$ denote a matched test condition, $S_{\mathrm{ref}}^{\left(j\right)}$ the waveform exported by the trusted black-box reference, $S_{C}^{\left(j\right)}$ the waveform exported by candidate implementation *C*, and $\Phi^{\left(j\right)}$ the diagnostic feature vector extracted from global, local-window, and event-level waveform comparisons. The predefined diagnostic outcome set is

(1) $$H=\{h_{\mathrm{none}},\;h_{\mathrm{init}},\;h_{\mathrm{event}},\;h_{\mathrm{history}},\;h_{\mathrm{expert}}\},$$

where $h_{\mathrm{none}}$ denotes no actionable fault, $h_{\mathrm{expert}}$ denotes insufficient or out-of-library evidence requiring expert review, and the remaining outcomes denote the three implementation mechanisms considered in this study. The diagnostic mapping is

(2) $$D:\left(\Phi^{\left(j\right)},C,u_{j}\right)\to h\in H.$$

This mapping performs closed-set mechanism selection using waveform features, event context, and candidate-source inspection, with explicit abstention when the evidence is insufficient. Unique causal reconstruction from waveform evidence alone lies outside the mapping.

Let C∈C denote a candidate source-level implementation of an EMT component model under diagnosis, where C is the admissible code space reachable through source-level edits. Let $S_{\mathrm{sim}}\left(C\right)$ denote the waveform generated by the simulator when executing *C*, and let $S_{\mathrm{ref}}$ denote the reference waveform produced by the black-box vendor component under the same test condition. The diagnosis-and-correction objective is formulated as

(3) $$C^{*}=\arg\min_{C\in C}L_{\mathrm{wave}}\left(S_{\mathrm{ref}},S_{\mathrm{sim}}\left(C\right)\right)\;s.t.\;\Gamma \left(C\right)=1,\;\Gamma_{\mathrm{beh}}\left(C\right)=1,\;A\left(C\right)=\mathrm{Pass},$$

Here, Γ(C)=1 indicates compile feasibility, $\Gamma_{\mathrm{beh}}\left(C\right)=1$ indicates that the case-specific behavioral-conformance condition on the waveform and event-level indicators of Section 3.3 is satisfied, and A(C) denotes the overall decision of the applicability-aware physics-and-discretization audit defined in Section 3.4, with A(C)∈{Pass,Reject,Expertreview}. A candidate correction is automatically accepted only when it compiles, satisfies the behavioral-conformance condition, and receives an overall Pass audit decision. A Reject outcome removes the candidate from the feasible repair set regardless of its waveform loss, whereas an Unresolved outcome of any applicable rule invokes expert review.

### 2.2. Overall Multi-Agent Architecture

**Figure 1:**
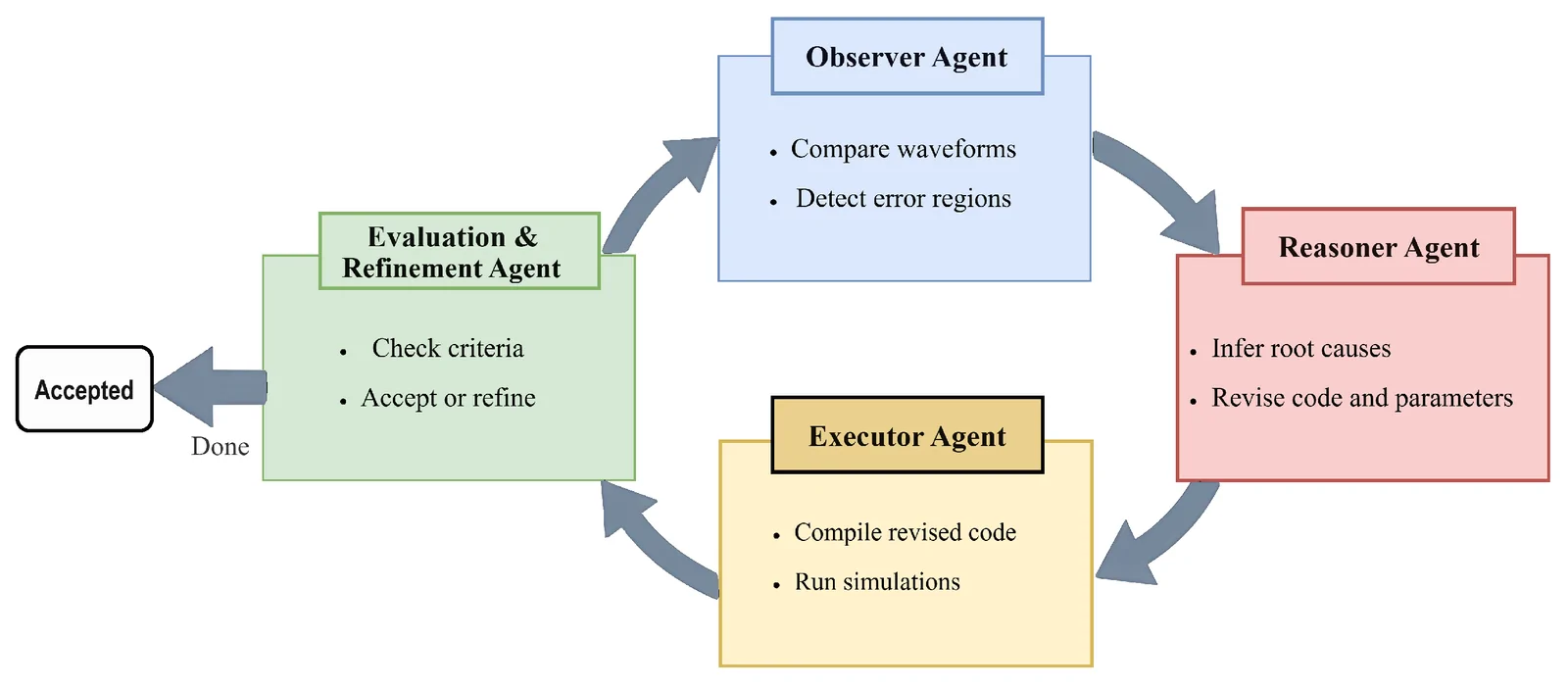
System architecture.

As shown in Figure 1, Observer, Reasoner, and Executor roles separate waveform analysis, mechanism inference, source editing, and simulator feedback [16]. A deterministic evaluation block applies the waveform/event conditions and physics-audit rules. The decomposition is used for traceability; the scientific object is the behavioral non-conformance diagnosis and constrained repair problem of Section 2.1.

### 2.3. Agent Roles

The Observer aligns waveforms and extracts global, local, and event features; the Reasoner maps them to EMT-specific source hypotheses; and the Executor applies edits and returns compilation, simulation, and waveform feedback [24,25,26]. The archived RTDS compilation, execution, and export steps were human-assisted, so the reported automation evidence concerns diagnosis, source editing, and closed-loop feedback rather than GUI reliability.

### 2.4. Rule-Based Evaluation-And-Refinement Stage

The evaluation-and-refinement stage closes the loop among the three LLM-based agent roles. After each candidate correction is executed, this stage checks the case-relevant acceptance condition: compile feasibility Γ(C)=1, the behavioral-conformance condition represented by $\Gamma_{\mathrm{beh}}\left(C\right)=1$ and evaluated using the waveform and event-level indicators of Section 3.3, and the overall audit decision A(C) defined in Section 3.4. A candidate is accepted only when the compile and behavioral gates are satisfied and no applicable audit rule returns Reject or Unresolved. Any Unresolved outcome routes the candidate to expert review; after a Reject or a failed behavioral gate, the diagnostic evidence is returned to the Reasoner for a further refinement iteration. This stage is implemented as deterministic rule checking rather than LLM generation, so that acceptance decisions within the multi-agent workflow remain reproducible and auditable.

## 3. Diagnosis-and-Correction Workflow and Physics-Constrained Evaluation

### 3.1. Platform Setup and Model Construction

This study adopts RTDS NovaCor as a representative commercial real-time EMT platform. The custom-component interface allows users to define component behavior through C source files. For benchmarking, each component model under diagnosis is compared against a vendor-provided black-box reference component under identical topology and excitation conditions.

### 3.2. Dual-Loop Diagnosis-and-Correction Workflow

**Figure 2:**
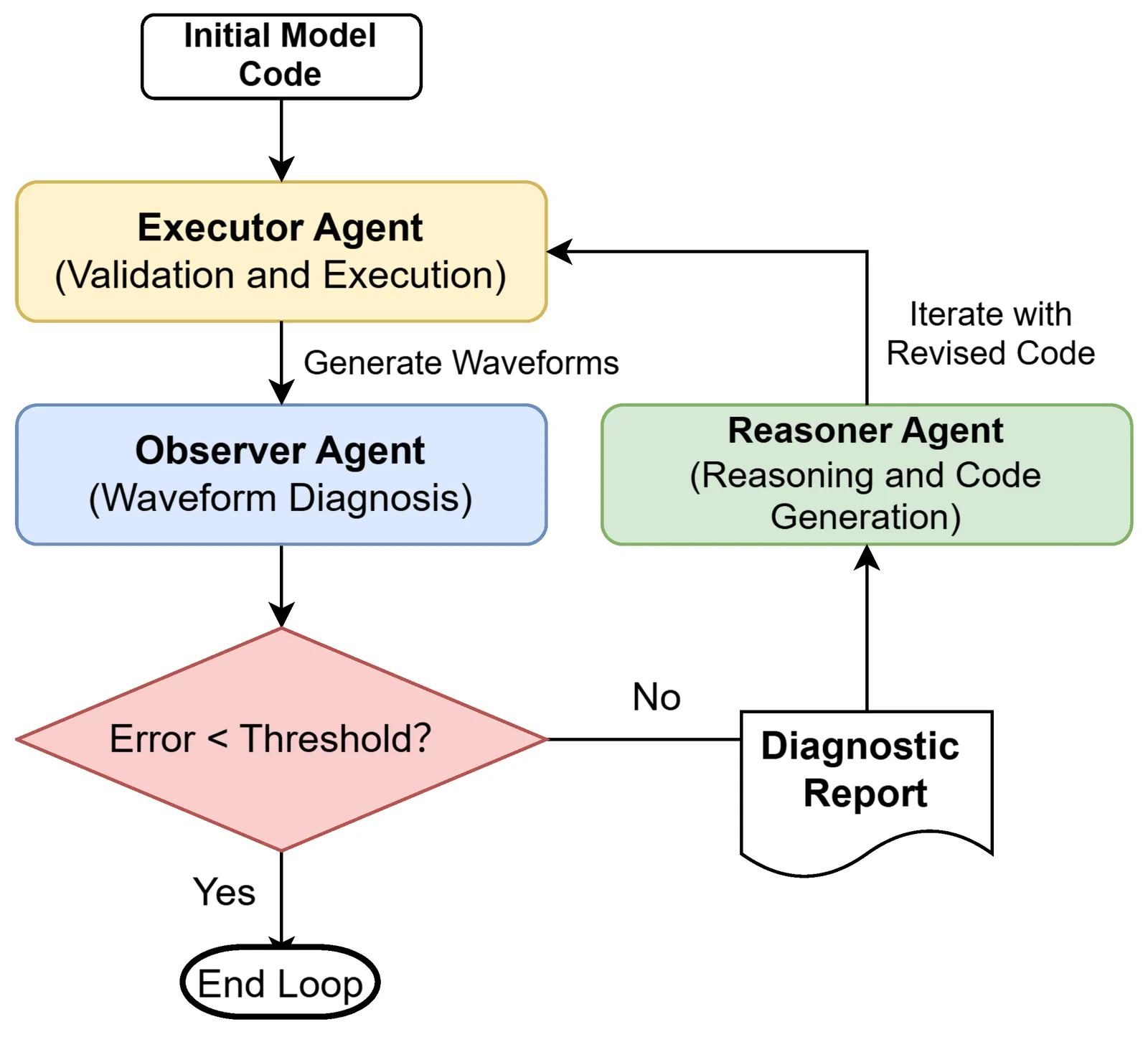
Diagnosis-and-correction workflow.

As illustrated in Figure 2, the process starts from an initial component implementation that may contain discretization, initialization, or event-transition faults. The inner loop corrects compiler-reported errors until Γ(C)=1, whereas the outer loop uses waveform-feature diagnostics, event-level indicators, and physics-audit feedback to guide structural source-code edits. This separation prevents syntactically valid but physically invalid code from being accepted solely because it compiles or reduces a scalar waveform error.

Each cycle first resolves compiler errors. After a successful build, the Observer aligns the candidate and reference waveforms and extracts ADM/FDM/GDM and event indicators. The Reasoner maps the dominant mismatch to a predefined fault class and proposes a targeted edit. Acceptance requires the case-specific behavioral condition and an overall audit Pass; Unresolved invokes expert review, while Reject or behavioral failure returns evidence for another iteration.

### 3.3. FSV-Inspired Waveform Metric and Event-Level Indicators

To overcome the limitations of using a scalar pointwise error alone, this study adopts two complementary waveform measures. The first is an FSV-inspired low/high-frequency decomposition metric, and the second is an event-level diagnostic indicator for highly localized implementation faults. This combination follows the FSV motivation of distinguishing amplitude-related and structural waveform differences [21,22,23,27].

The reference waveform $S_{\mathrm{ref}}$ and the candidate waveform $S_{\mathrm{sim}}\left(C\right)$ are first aligned on a common time axis. For paired-file comparisons, the candidate waveform is interpolated onto the reference time axis over the common time interval. No amplitude normalization is applied before decomposition; the reference RMS is used only as the normalization scale in the metric calculation.

Each aligned waveform x(k) is decomposed into a low-frequency component and a high-frequency residual using a centered moving-average filter with a window length of 21 samples:

(4) $$x_{\mathrm{low}}\left(k\right)={\mathrm{MA}}_{21}\left\{x\left(k\right)\right\},\;x_{\mathrm{high}}\left(k\right)=x\left(k\right)-x_{\mathrm{low}}\left(k\right).$$

For a reference waveform r(k) and a candidate waveform c(k), the normalization scale is

(5) $$s=\max\left(\sqrt{\frac{1}{N}\sum_{k=1}^{N}r^{2}\left(k\right)},\;10^{-12}\right).$$

The amplitude difference measure (ADM), feature difference measure (FDM), and global difference measure (GDM) are

(6) $$\mathrm{ADM}=\frac{\sqrt{\frac{1}{N}\sum_{k=1}^{N}{\left(r_{\mathrm{low}}\left(k\right)-c_{\mathrm{low}}\left(k\right)\right)}^{2}}}{s},$$

(7) $$\mathrm{FDM}=\frac{\sqrt{\frac{1}{N}\sum_{k=1}^{N}{\left(r_{\mathrm{high}}\left(k\right)-c_{\mathrm{high}}\left(k\right)\right)}^{2}}}{s},$$

(8) $$\mathrm{GDM}=\sqrt{{\mathrm{ADM}}^{2}+{\mathrm{FDM}}^{2}}.$$

ADM reflects low-frequency magnitude, offset, and trend deviations, whereas FDM reflects local slope changes, fast transients, and high-frequency structural differences. This is a transparent FSV-inspired implementation, not a certified IEEE Std 1597.1 FSV tool.

The event-level indicator is used when the fault is highly localized in time. For the pointwise error e(k)=r(k)−c(k), the maximum instantaneous error is

(9) $$E_{\max}=\max_{k}\left|e\left(k\right)\right|,$$

and the corresponding time is denoted by $t(E_{\max})$. A divergence event is reported when |e(k)| exceeds a case-specific threshold $\tau_{e}$ derived from a matched nominal reference–candidate residual where available, or from a stated engineering acceptance tolerance otherwise. For localized faults, the same ADM/FDM/GDM decomposition can also be applied within a short window centered on the divergence instant. This local-window analysis complements the global metric and event-level maximum error.

### 3.4. Physics-Audit Constraints

A low global waveform error alone does not guarantee physical admissibility. The framework therefore applies rule-based static physics-audit constraints to generated source-code changes.

Each audit rule $i\in \{1,\ldots ,N_{r}\}$, $N_{r}=6$, is evaluated with an explicit applicability condition and returns a four-valued outcome

(10) $$A_{i}\left(C\right)\in \left\{\mathrm{Pass},\;\mathrm{Reject},\;N/A,\;\mathrm{Unresolved}\right\},$$

where N/A indicates that the component does not contain the state, event, or declared constraint addressed by the rule, and Unresolved indicates that admissibility cannot be decided from the source code and the available component specification alone. The overall audit decision is

(11) $$A\left(C\right)=\left\{\begin{array}{cc} \mathrm{Reject}, & \exists \;i:\;A_{i}\left(C\right)=\mathrm{Reject},\\ \mathrm{Expert}\;\mathrm{review}, & ∄\;i:\;A_{i}\left(C\right)=\mathrm{Reject}\;\wedge \;\exists \;i:\;A_{i}\left(C\right)=\mathrm{Unresolved},\\ \mathrm{Pass}, & \mathrm{otherwise}. \end{array}\right.$$

The audit is a conservative admissibility screen rather than a proof of physical correctness: a candidate is automatically accepted only when no applicable rule returns Reject or Unresolved.

A repairable violation, such as an initialization-closure fault, still produces a Reject outcome for the current candidate; the rule may additionally provide a targeted repair suggestion for the next iteration.

The six audit rules and their default outcomes are summarized in Table 1.

Component-specific rule applicability is summarized in Table 2.

InvSqrt uses a first-step latch but otherwise follows an algebraic control path. VARRL parameter commands are treated as continuous parameter updates, so their temporal consistency is evaluated under R2 rather than the switching-event rule R3. In the offline challenge-set evaluation, the rules and component contracts were frozen and hashed before any candidate was evaluated. The audit uses structured lexical and data-flow checks against the frozen contracts; it does not use candidate file names or identities as decision inputs, and repeated executions produce byte-identical outputs. Every Reject and Unresolved outcome cites the applicable rule identifier and source-code line evidence. When a contract is absent or ambiguous, the rule returns Unresolved rather than a forced decision.

### 3.5. Fault Classification and Metric Interpretation

Table 3 links diagnostic evidence to the outcomes in H and the corresponding actions. The mapping is a predefined closed-set scheme; unresolved or out-of-library evidence requests expert inspection rather than a forced correction.

The diagnostic outcome set is denoted by $H=\{h_{\mathrm{init}},h_{\mathrm{event}},h_{\mathrm{history}},h_{\mathrm{none}},h_{\mathrm{expert}}\}$.

The ADM/FDM/GDM indices provide a global low/high-frequency decomposition of waveform mismatch and are useful for distributed residual errors. However, the breaker case shows that a physically important implementation fault may occur at one switching sample and can be under-represented by any globally averaged metric. Event-level maximum error and divergence time are therefore reported together with global metrics for localized faults.

## 4. Experimental Setup

The study uses RTDS NovaCor as the real-time EMT platform. The Reasoner role, and the LLM-assisted parts of the Observer and Executor roles, were instantiated using the DeepSeek-V3.2-Reasoner model through an API interface with temperature set to 0.0 and maximum generation length set to 8192 tokens. The automation wrapper was based on mini-sweagent version 1.17.4. Three components were selected because they expose distinct fault mechanisms: initialization inconsistency, switching-event inconsistency, and history-state inconsistency.

The three cases were retained from component-conversion and verification records associated with the engineering project rather than synthesized as artificial waveform perturbations for this paper. They are used as project-specific, mechanism-representative cases.

Their diagnostic difficulty differed by mechanism. The InvSqrt fault affected only the first simulation sample and could pass routine visual inspection of steady-state waveforms. The breaker fault produced two nonzero differences among 20,000 samples, with the dominant error at a single switching instant; global metrics dilute such temporally sparse effects. The VARRL fault involved coupled filtering, Norton-equivalent, and history-update paths whose observable effects depended on the applied parameter commands; the controlled source-variant paths were dormant under constant parameters and activated by specific command-step events. These characteristics illustrate why single-condition conformance testing and global scalar metrics may miss latent implementation faults.

### 4.1. Implementation Details and Reproducibility

The diagnosis-and-correction workflow was implemented as a closed-loop process in which waveform analysis and source-code editing were automated. In the archived trajectories analyzed in this paper, RTDS compilation, simulation execution, and waveform export used a human-assisted interaction step rather than fully autonomous GUI control. Accordingly, the recorded trajectories evaluate the diagnostic and repair loop but not GUI-control reliability.

The same waveform-export format, comparison procedure, and audit-rule categories were used across the cases. Event-level thresholds were case-specific and were derived from matched nominal residuals where available or from stated engineering acceptance tolerances otherwise. RMSE was retained only as an auxiliary diagnostic signal and was not used as a sole stopping criterion. The final acceptance condition requires compile feasibility Γ(C)=1, the case-relevant waveform/event indicators to satisfy $\Gamma_{\mathrm{beh}}\left(C\right)=1$, an overall audit decision A(C)=Pass, and no newly detected fault signature.

The temperature setting of 0.0 reduces generation variability but does not guarantee bit-level determinism of an API-served LLM. The RTDS project files, platform-specific benchmark models, and source-code implementation details are subject to license and project restrictions. All waveform metrics reported in this paper were recomputed offline from saved CSV files using the same in-house waveform-metric script with a 21-sample moving-average window, so that ADM, FDM, GDM, RMSE, and event-level indicators are defined consistently across the three cases.

For the InvSqrt case, the initial comparison showed a startup single-sample deviation of 0.5 at t=0, with RMSE 0.007906 and RMS relative difference 1.581%. For the single-phase breaker case, the dominant divergence occurred at t=0.50075 s, where the maximum instantaneous current error was 0.950469; the global RMSE was 0.006721 and the RMS relative difference was 0.042%. For the VARRL case, retained diagnostic logs recorded initial RMS relative differences of 8.762%, 18.522%, and 16.858% for the three phase-paired black-box and candidate channels, respectively.

Table 4 summarizes the correction results for the three project cases.

### 4.2. Extended Multi-Condition Validation Within the VARRL Case

The VARRL implementation was evaluated using five separate VARRL test branches driven by matched source blocks within one RSCAD case: the RTDS black-box reference (REF), the retained corrected white-box implementation (C0), and three controlled executable source variants derived from the same VARRL component. X1 used inconsistent inductance snapshots between the Norton-conductance and history-current calculations; X2 delayed the Norton-equivalent parameters by one simulation step; and X3 bypassed the specified inductance rate limiter. The variants modify executable CBuilder source code; the exported waveforms are unmodified solver outputs.

The five branches were evaluated under four matched operating conditions at a 50 μs time step: constant *R* and *L*, an R-only command step, an L-only command step, and a simultaneous R/L command step applied at t=2.0 s. The constant-parameter condition was recorded for 0.2 s (4000 samples) because no command event was present and its purpose was to verify that the altered source paths remained dormant; the three command-step conditions were recorded for 2.5 s (50,000 samples) to include the event and the subsequent transition. Dynamic-condition metrics are evaluated on the 200 ms transition window t∈[2.0,2.2] s (4001 samples).

The node labels in this extended parallel-branch RSCAD case are local to that case and differ from the labels used in the previously retained VARRL waveform files: N1–N3 correspond to REF, N4–N6 to C0, N7–N9 to X1, N10–N12 to X2, and N13–N15 to X3. These labels should not be interpreted as common electrical buses across the two RSCAD cases.

All comparisons use the metric definitions of Section 3.3 with the same 21-sample moving-average decomposition. Within each comparison pair, the first-listed waveform provides the RMS normalization scale: REF-to-C0 and REF-to-variant comparisons are normalized by the REF RMS in the evaluated window, whereas C0-to-variant comparisons are normalized by the C0 RMS. REF-to-variant and REF-to-C0 values therefore share the same normalization and can be compared directly, whereas C0-to-variant values are used to isolate the direct effect of each source modification.

For this controlled within-case evaluation, a source path is reported as *activated* under a condition only when both the transition-to-pre-event C0-to-variant GDM ratio and the corresponding maximum-error ratio exceed $10^{2}$.

### 4.3. Offline Cross-Component Audit Challenge Set

To evaluate whether the applicability-aware audit of Section 3.4 can reject inadmissible source modifications without rejecting legitimate alternative implementations, an offline ten-candidate source-code challenge set was constructed across three component structures: the algebraic InvSqrt block, the event-driven breaker, and the history-state-based VARRL component. Three candidates are designed semantics-preserving refactorings of the retained corrected implementations. Six candidates each introduce one documented inadmissible mechanism targeting one audit-rule category; the VARRL X1 and X3 variants are included in this group. The remaining candidate is the VARRL X2 one-step parameter-delay variant, for which the intended time-index convention is underspecified and an abstention is required.

The audit rules and component contracts were frozen and hashed before evaluation. All ten candidates compiled under the target toolchain (RSCAD FX 2.3 CBuilder; RTDS Technologies Inc., Winnipeg, MB, Canada); the six inadmissible candidates are therefore compilable-but-inadmissible implementations whose defects are invisible to the compile-feasibility gate. The audit was executed by a deterministic rule checker operating on blinded file names, containing no candidate-specific logic as checked by unit tests, and citing line-level source evidence for every Reject and Unresolved outcome; repeated executions produced byte-identical results. Candidate designations derive from documented single-mechanism construction and were not independently re-annotated. Compilation establishes CBuilder compatibility, not dynamic correctness; runtime behavior is documented only for C0 and X1–X3 in the within-case VARRL evaluation.

### 4.4. Retained Trajectory Evidence

Four retained successful trajectories were available for InvSqrt and the breaker. They show that the physics context changed the proposed repair from a generic delay to first-step gating and reduced recorded API calls from 47 to 34. In both breaker trajectories, the first candidate worsened RMSE before waveform feedback corrected the state-update ordering. Because unsuccessful runs were not systematically archived, the complete trajectory table can be made available from the corresponding author upon reasonable request; accordingly, no success rate or architecture comparison is inferred.

## 5. Benchmark Demonstrations on Representative EMT Fault Classes

The three demonstrations cover start-up initialization faults, switching-event faults, and history-state update faults. RTDS denotes the black-box reference branch, and SEPRI denotes the candidate white-box model under diagnosis.

### 5.1. Case I: Inverse-Square-Root Component (InvSqrt)

**Figure 3:**
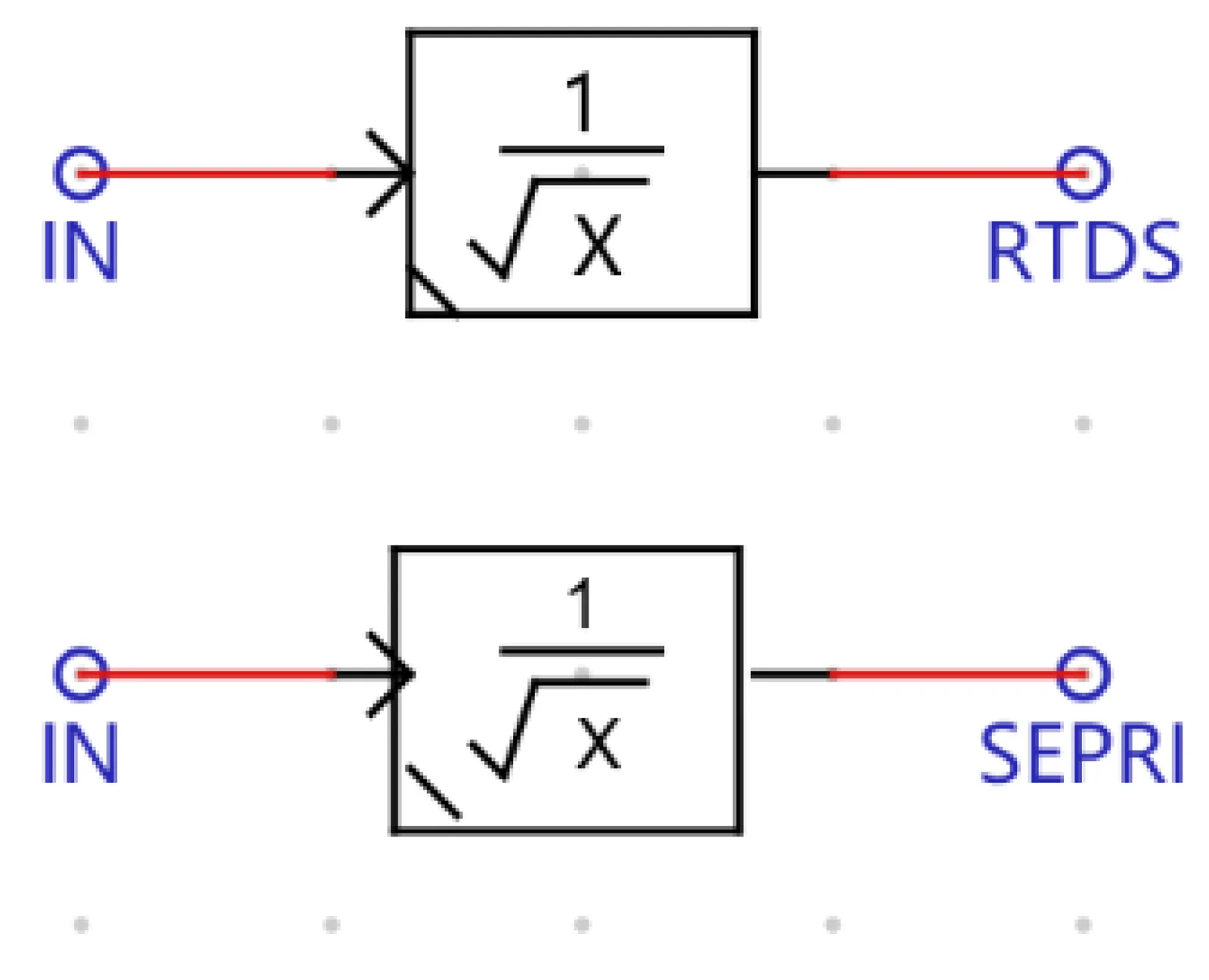
InvSqrt test circuit topology.

InvSqrt is an inverse-square-root control component. The same input signal is applied to the black-box reference and the component model under diagnosis. The corresponding circuit topology is shown in Figure 3.

The fault was caused by an initialization–timing mismatch. At t=0, the original code evaluated $1/\sqrt{\mathrm{INP}}$ before the input state was fully established, producing a non-physical startup pulse. The corrective action used first-step gating and static-state closure.

**Figure 4:**
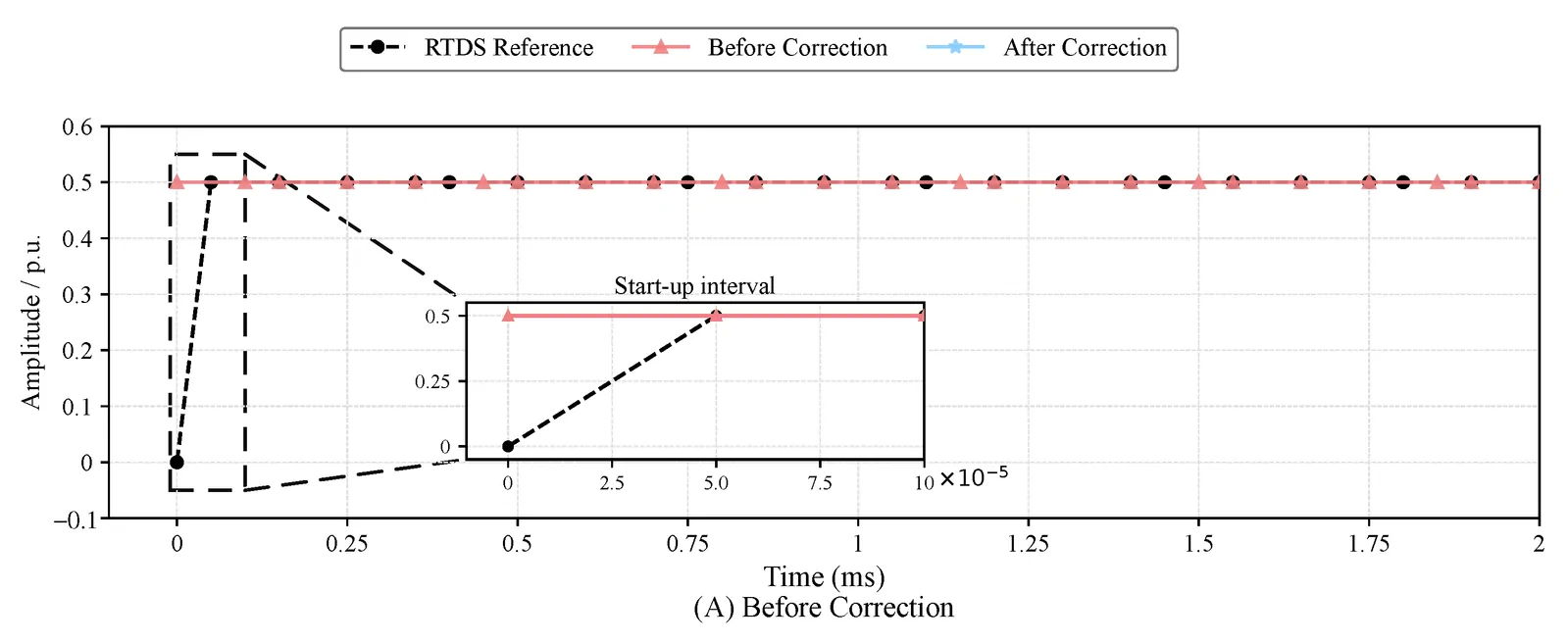

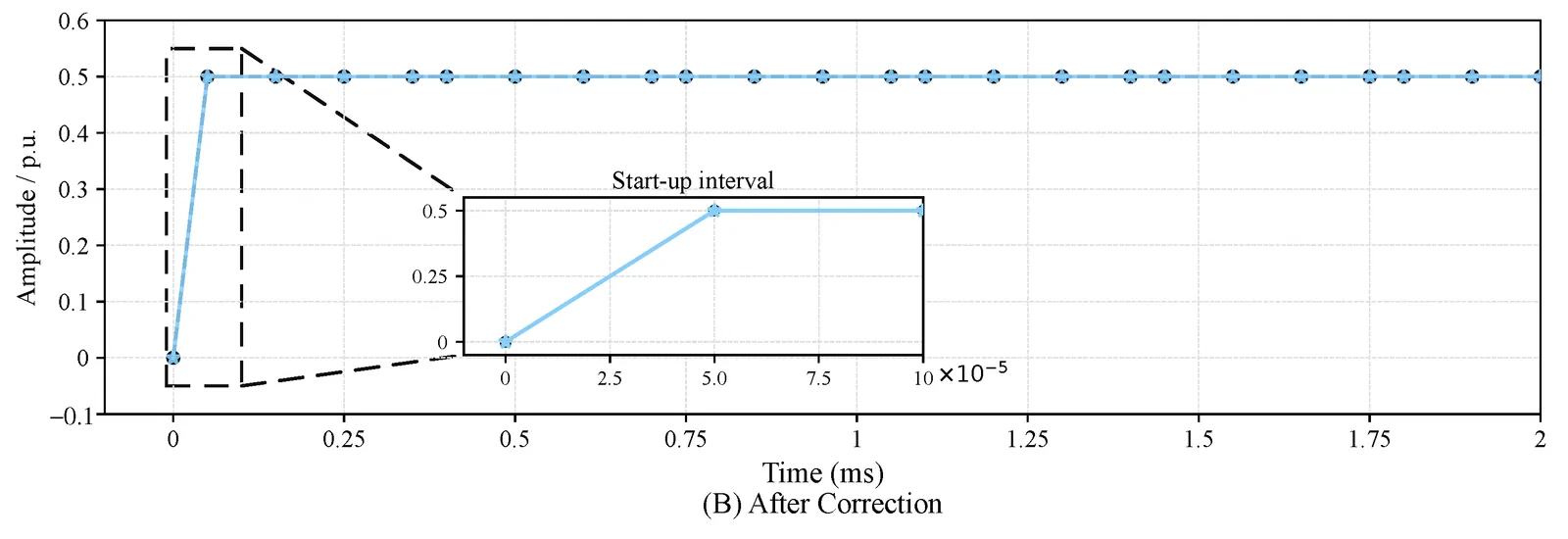
InvSqrt waveform comparison. The startup pulse before correction is localized at the first sampling instant, and the corrected component matches the RTDS reference within exported waveform precision.

Before correction, the FSV-inspired GDM was 0.023705, with ADM 0.016939 and FDM 0.016583. The dominant error was a single-sample startup deviation of 0.5 at t=0. The record contains 4000 samples at a 50 μs time step (0.2 s total); only the first sample differed, with the remaining 3999 samples agreeing within exported precision. After first-step gating, the candidate output matched the reference within exported CSV precision, and the GDM, ADM, FDM, RMSE, and maximum error were all reduced to zero. After its first-step latch is set, InvSqrt follows an algebraic control path; exact pointwise agreement is therefore expected after the initialization correction. The waveform comparison is shown in Figure 4.

### 5.2. Case II: Single-Phase Breaker

**Figure 5:**
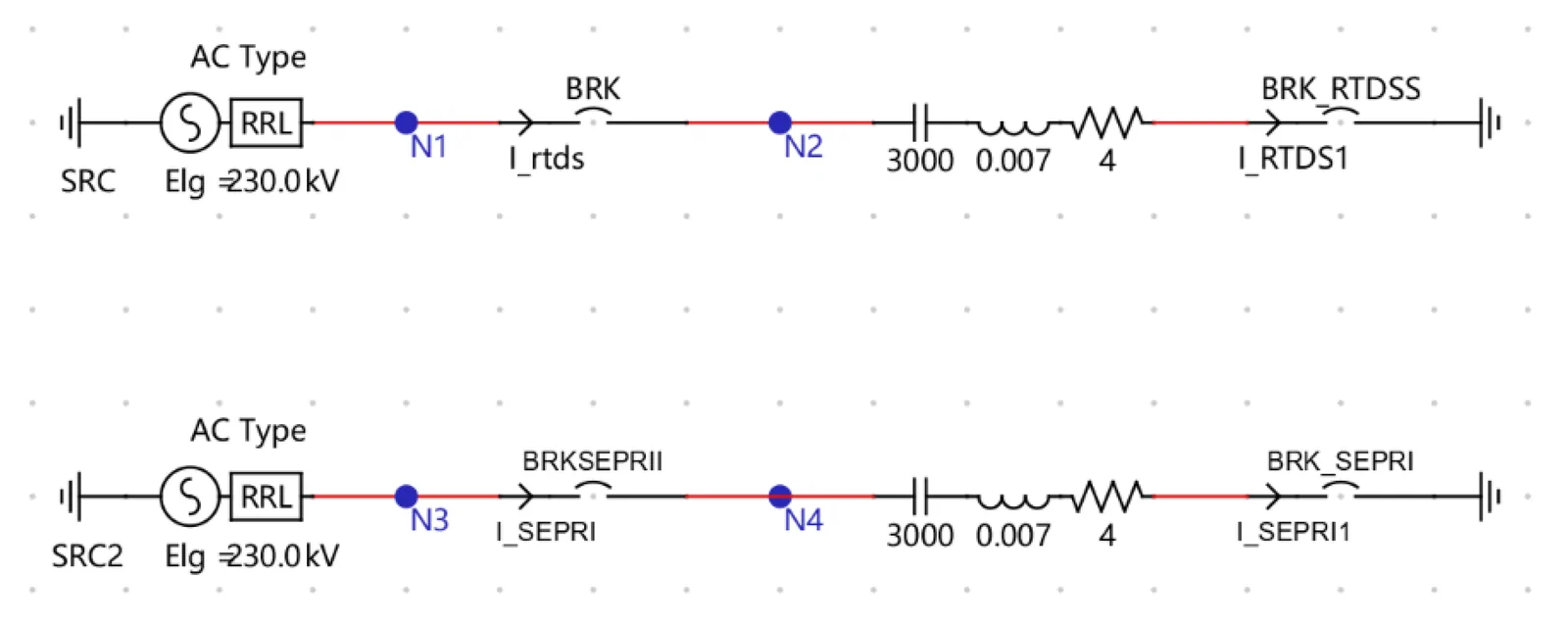
Breaker test circuit topology.

The breaker case tests event logic around current-zero interruption. The upper branch uses the black-box reference breaker, and the lower branch uses the component model under diagnosis. The source voltage is 230.0 kV, with $r_{\mathrm{off}}=1.0\times 10^{6}\;\Omega$, $r_{\mathrm{on}}=0.1\;\Omega$, and holdi=0kA. The test topology is shown in Figure 5.

The initial breaker model showed a localized event-state mismatch near the current-zero switching instant. Parameter tuning could not remove the event-level mismatch, so the correction shifted to source-level state-machine restructuring and history-state smoothing.

Before correction, only two of the 20,000 exported samples exhibited nonzero differences. The dominant switching-event discrepancy occurred at t=0.50075 s with an absolute current error of 0.950469 A, occupying 0.005% of the record; the secondary difference, at t=0.30005 s, was only $6.12\times 10^{-6}$ A. Thus, only one sample exceeded $10^{-5}$ A. The global GDM was only 0.000423 because the dominant error was temporally sparse and diluted by full-record averaging. Therefore, the event-level maximum error is the primary indicator for this case. After event-transition restructuring and history-state correction, the switching-instant deviation was eliminated within exported CSV precision.

**Figure 6:**
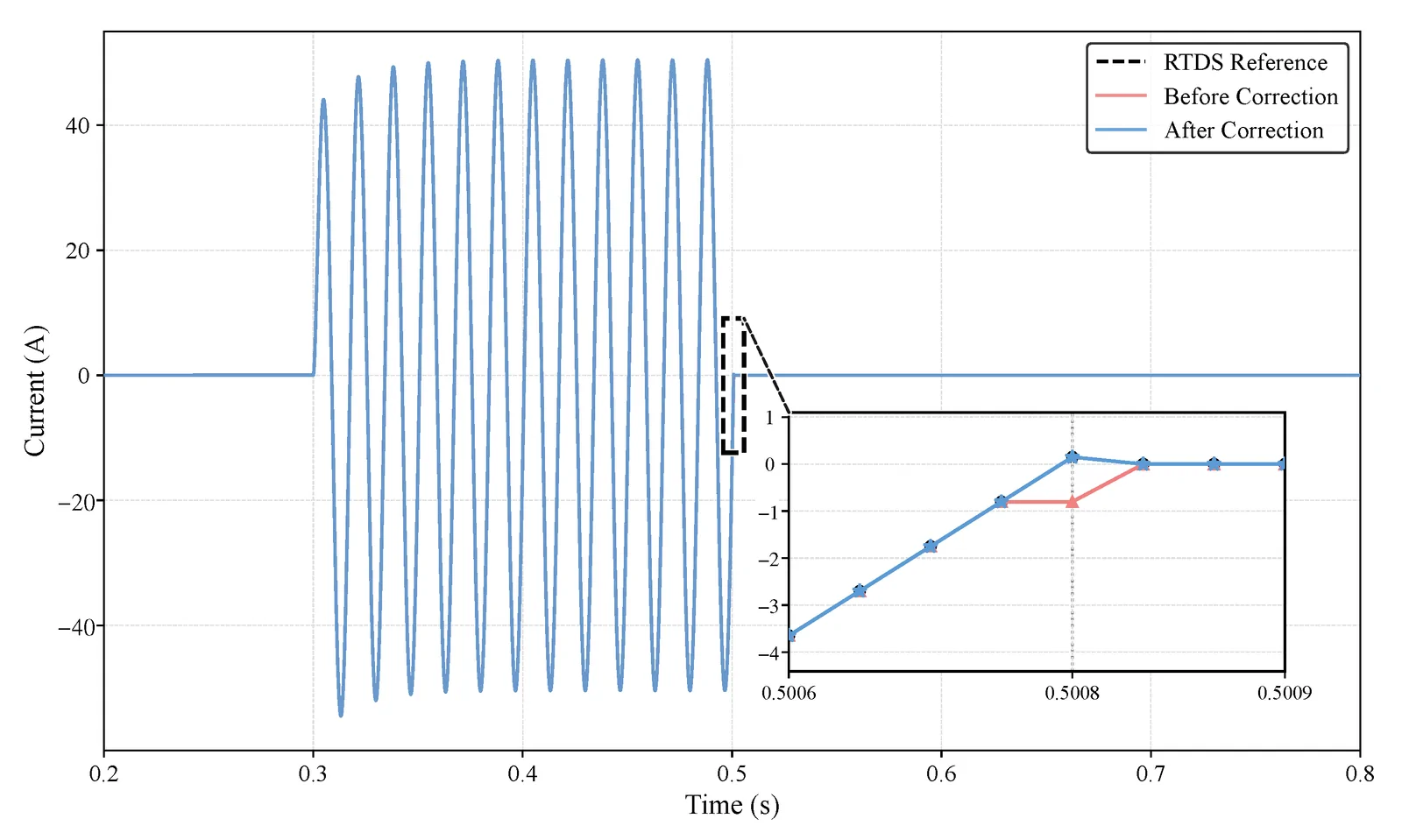
Breaker waveform comparison. The inset highlights the localized switching-instant mismatch near t=0.50075 s before correction and its removal after correction.

The secondary pre-correction difference at t=0.30005 s should not be confused with the much larger transient spike introduced by the rejected blind-run candidate near the same instant (Section 4.4). The pre- and post-correction breaker waveforms are shown in Figure 6.

A local-window analysis confirms that FDM remains meaningful at the correct time scale. A 101-sample window centered on t=0.50075 s was extracted, and the same 21-sample decomposition was applied. As shown in Table 5, FDM increases from 0.000412 globally to 0.005180 locally, about 12.6× larger. This confirms that the structural mismatch is concentrated near the switching event rather than distributed over the whole record.

### 5.3. Case III: Variable Impedance Component (VARRL)

The VARRL case compares the black-box reference and candidate implementations under the same 230.0 kV source and control input. In the original single-branch waveform exports, the black-box channels N4–N6 are paired phase-wise with the candidate channels N1–N3: N4 corresponds to N1, N5 to N2, and N6 to N3. These channel labels belong to separate waveform exports and do not denote common electrical buses. The extended parallel-branch case uses the different node mapping defined in Section 4.2. The main parameters are $R_{init}=1.0\;\Omega$, $R_{min}=0.01\;\Omega$, $R_{max}=100.0\;\Omega$, $L_{init}=0.05\;H$, $L_{min}=0.01\;H$, $L_{max}=0.1\;H$, and dL=0.5H/s.

#### 5.3.1. Discretization Background

The VARRL component represents a three-phase controllable series RL branch. For each phase, the continuous-time branch relation is

(12) $$v\left(t\right)=R\left(t\right)i\left(t\right)+L\left(t\right)\frac{di(t)}{dt}.$$

In the custom-component implementation, the branch is interfaced with the EMT network through a Norton-equivalent form. Using trapezoidal integration, the branch current at time step *k* can be written as

(13) $$i^{\left(k\right)}=G^{\left(k\right)}v^{\left(k\right)}+I_{\mathrm{hist}}^{\left(k\right)},$$

where the equivalent conductance is

(14) $$G^{\left(k\right)}=\frac{1}{R_{f}^{\left(k\right)}+2L_{f}^{\left(k\right)}/\Delta t}.$$

**Figure 7:**
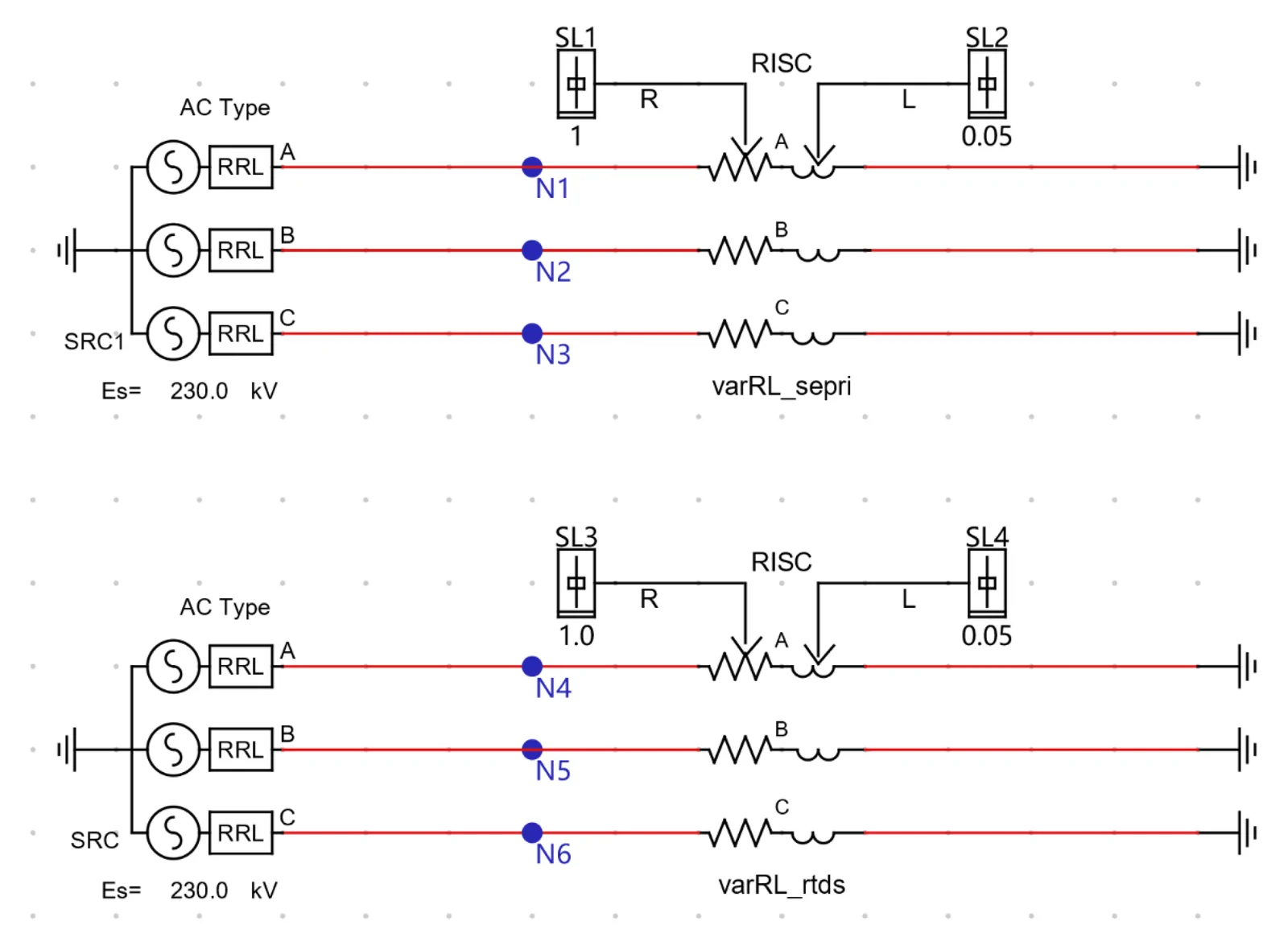
VARRL test circuit topology.

A representative history-current term is

(15) $$I_{\mathrm{hist}}^{\left(k\right)}=G^{\left(k\right)}\left[v^{\left(k-1\right)}+\left(\frac{2L_{f}^{\left(k\right)}}{\Delta t}-R_{f}^{\left(k\right)}\right)i^{\left(k-1\right)}\right],$$

with the sign convention following the branch voltage and current orientation used in the component implementation. This expression shows why previous-step voltage, previous-step current, and the equivalent parameters must remain consistently latched until the current-step Norton update is completed. The corresponding test circuit is shown in Figure 7.

The commanded resistance and inductance are smoothed before entering the Norton update. For a generic command $x^{\left(k\right)}$ and filtered value $y^{\left(k\right)}$, a first-order low-pass filter

(16) $$T\frac{dy(t)}{dt}+y\left(t\right)=x\left(t\right)$$

is discretized by the trapezoidal rule as

(17) $$y^{\left(k\right)}=\frac{\left(1-\alpha \right)y^{\left(k-1\right)}+\alpha \left(x^{\left(k\right)}+x^{\left(k-1\right)}\right)}{1+\alpha },\;\alpha =\frac{\Delta t}{2T}.$$

The filtered inductance is further constrained by the rate limiter

(18) $$\left|L_{f}^{\left(k\right)}-L_{f}^{\left(k-1\right)}\right|\leq dL\Delta t,$$

and both $R_{f}$ and $L_{f}$ are clipped to their admissible physical ranges. These coupled operations explain why the VARRL case is more sensitive than the InvSqrt and breaker cases: parameter filtering, rate limiting, equivalent conductance update, and history-current latching occur in the same discrete-time execution path.

**Figure 8:**
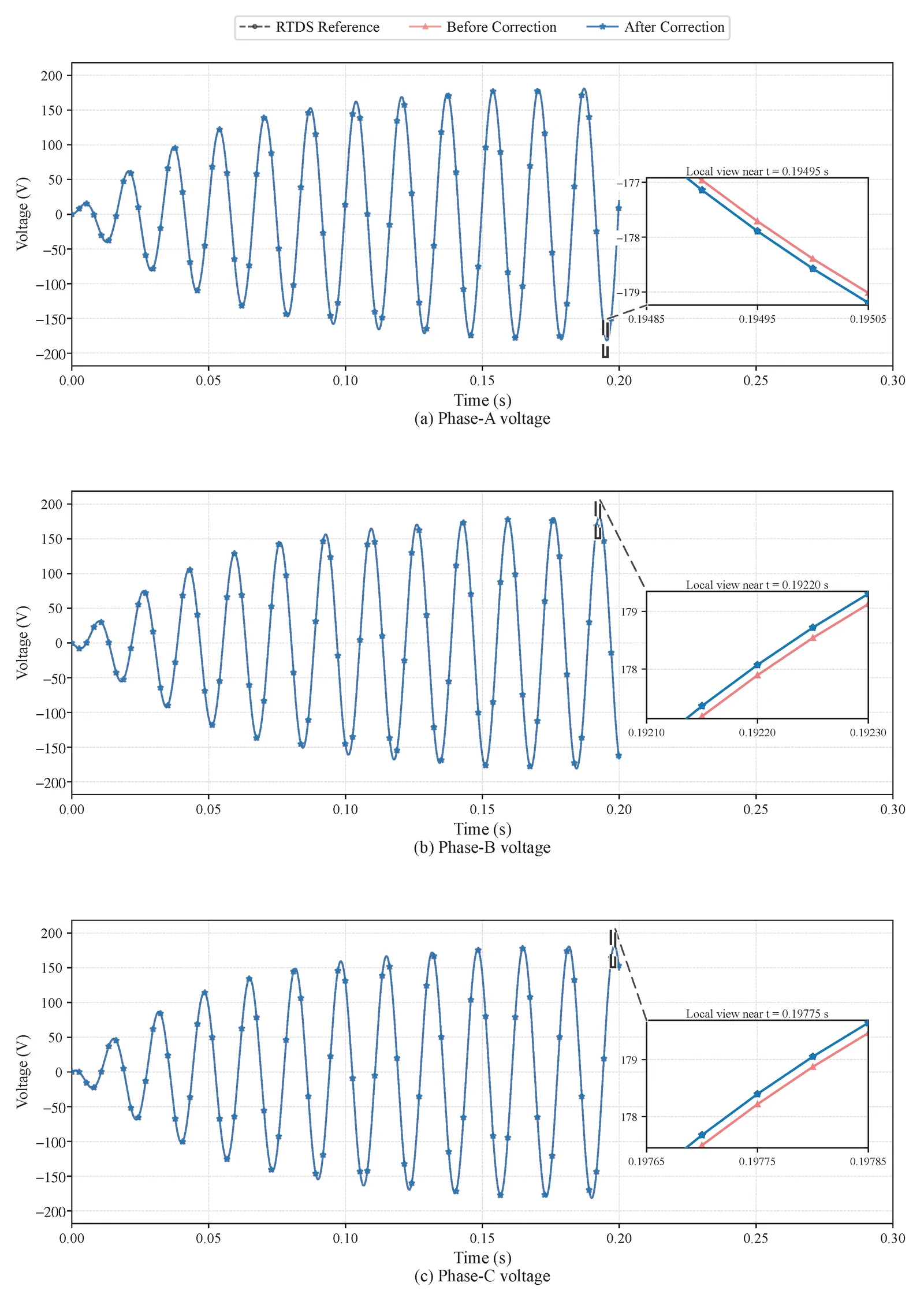
VARRL waveform comparison for the three phase-paired channels. Black-box channels N4–N6 are compared with candidate channels N1–N3, respectively, and the insets show local voltage views near the dominant pre-correction mismatch intervals.

The VARRL mismatch came from coupled parameter filtering, rate limiting, Norton-equivalent conductance updates, and history-current re-latching. A dead-zone shortcut reduced local RMSE but was rejected because it reset the history current and violated inductive current continuity. The accepted correction combined cold-start history-state latching with update-order correction. The resulting waveform comparison is shown in Figure 8.

After correction, the dominant transient mismatch was reduced but not fully eliminated. The residual comparison pairs black-box channels N4–N6 with candidate channels N1–N3, respectively. The three phase-paired RMS relative differences were 0.098%, 0.099%, and 0.099%. The residual FSV-inspired GDM was 0.000979, with ADM 0.000979 and FDM 0.000007. This result is reported as engineering-usable mitigation rather than complete waveform elimination.

#### 5.3.2. Within-Case Multi-Condition Source-Variant Evaluation

As an extension of the VARRL case, the retained corrected implementation C0 and the three controlled source variants X1–X3 were evaluated under the four matched operating conditions defined in Section 4.2. The variants are controlled mechanism probes within Case III, not additional project-derived component cases.

Under the constant-parameter condition, all three variants remained behaviorally dormant, with phase-mean C0-to-variant GDM values on the order of $10^{-10}$, limited by CSV export precision. The parameter-step conditions produced a mechanism-consistent activation matrix (Table 6). Under the R-only step, only X2 was activated, while X1 and X3 remained at the export-precision floor, constituting mechanism-specific negative controls. Under the L-only and coupled R/L steps, X1 and X3 were also activated, consistent with their inductance-related source modifications. The L-only X1 and R-only X2 variants produced transition-window RMS relative differences of only 0.02749% and 0.01831%, respectively. Both are executable controlled source variants whose waveform effects are substantially smaller than the X3 deviations. X3 produced substantially larger deviations (0.895% under L-only and 12.82% under R + L) because bypassing the declared inductance-rate constraint changes the component transition semantics.

Activation, however, did not always imply separability from the matched reference discrepancy. Under the L-only condition, the C0-to-X2 RMS relative difference was only 0.000551%, and the REF-to-X2 GDM was essentially equal to the matched REF-to-C0 GDM ($R_{\mathrm{sep}}=\mathrm{GDM}\left(\mathrm{REF},X2\right)/\mathrm{GDM}\left(\mathrm{REF},C0\right)\approx 1.00$); this activated-but-inseparable case is assigned to the unresolved/expert-review outcome $h_{\mathrm{expert}}$ rather than being forced into a known class. Under the R-only condition, X2 exceeded the matched baseline by only 1.75%, a weak descriptive margin. Notably, under the coupled R/L condition, X1 produced a *smaller* reference-based GDM (0.011782) than the retained corrected implementation C0 (0.012893), although X1 violates the same-step parameter-snapshot contract. Proximity to an opaque reference waveform alone, therefore, cannot adjudicate the correctness of the internal discretization semantics. The audit gate of Section 3.4 addresses this case.

$R_{G}$ is the transition-to-pre-event C0-to-variant GDM ratio; a source path is reported as activated only when both $R_{G}$ and the maximum-error ratio exceed $10^{2}$ (an operational within-case rule, not a universal threshold). Pre-event differences are limited by CSV export precision, so activation ratios indicate orders-of-magnitude elevation above the precision floor. REF-to-variant and REF-to-C0 GDM values share the same REF-based normalization. The Interpretation column combines the behavioral separability assessment with the audit outcome of Table 7; separability values alone do not determine audit decisions.

The matched REF–C0 discrepancy was itself dependent on both the operating condition and the evaluation window. The original constant-parameter record produced a full-record GDM of 0.000979 and an RMS relative difference of 0.09854%. For the L-only, R-only, and simultaneous R/L conditions, the full-record RMS relative differences were 0.10053%, 0.31078%, and 0.38810%, respectively, and the corresponding 200 ms transition-window values increased to 0.13967%, 1.03943%, and 1.29074%. These larger transition-window values do not contradict the previously reported constant-condition residual: the command-step events excite the coupled filtering, Norton-equivalent, and history-update path, and the event-local window is not diluted by the quiescent portion of the record. Because RTDS execution is deterministic, these matched REF–C0 values are condition-specific nominal model-to-reference discrepancies rather than a stochastic solver-noise distribution. This condition and window dependence indicate that no single scalar threshold can universally separate an implementation fault from the matched model-to-reference discrepancy; condition-matched baselining is therefore required.

These are source-admissibility outcomes only, not overall repair-acceptance decisions; overall acceptance additionally requires compile feasibility and behavioral conformance (Equation (3)). X1–X3 are included in the broader offline challenge set (Table 8); the present table links their source-audit decisions to the matched RTDS waveform results.

### 5.4. Cross-Component Offline Audit Challenge-Set Results

Table 8 summarizes the outcomes for the protocol defined in Section 4.3. All ten candidates compiled successfully. The deterministic audit returned Pass for the three designed semantics-preserving refactorings, Reject for the six single-mechanism inadmissible candidates—one per audit-rule category, each with line-level source evidence—and Expert review for the underspecified one-step-delay candidate. The corresponding VARRL outcomes are consistent with Table 7: the C0-derived refactoring received Pass, while X1, X2, and X3 received Reject, Expert review, and Reject, respectively. Rule applicability followed the frozen component contracts. For example, the discretization-and-snapshot rule is N/A for the algebraic InvSqrt block, and the declared-constraint rule is N/A for the breaker, whose specification declares no limiter. The parser status was complete for all 60 candidate–rule evaluations.

Two results clarify the audit’s intended role. First, the Pass outcomes for all three designed refactorings indicate that the rules respond to the stated semantic contracts rather than to the specific code organization of the corrected implementations. Second, because all six inadmissible candidates compile, none of the six Reject outcomes could have been produced by the compile-feasibility gate alone. The challenge set provides preliminary offline evidence for the six exercised rule categories; it does not estimate population-level false-acceptance or false-rejection rates.

Each Reject and the Unresolved rule outcome cites line-level source evidence in the final audit matrix, which can be made available from the corresponding author upon reasonable request. Rules and contracts were frozen and hashed before evaluation, and repeated executions produced byte-identical outputs. Candidate designations derive from documented single-mechanism construction and were not independently re-annotated.

### 5.5. Waveform-Metric Sensitivity Analysis

A supplementary offline perturbation analysis characterizes the waveform metrics using amplitude scaling, sample shift, a single-sample spike, high-frequency ripple, and a first-sample offset. With the 21-sample decomposition of Section 3.3, amplitude scaling primarily increased ADM, whereas spikes, ripple, and first-sample offsets primarily increased FDM; a global one-sample breaker shift mainly affected ADM. The complete perturbation table can be made available from the corresponding author upon reasonable request. This analysis describes metric response only; executable source-level evidence is reported in Section 5.3.2 and Section 5.4.

## 6. Discussion

### 6.1. Comparison with Existing Correction Paradigms

The method differs from PSO-based calibration and deep-reinforcement-learning-based identification, which are useful when model equations are correct and only parameters need tuning. Here, the dominant errors are code-logic faults, including wrong initialization order, unstable switching logic, and inconsistent history-state updates. The method also differs from direct LLM code generation because compile checks, waveform-feature feedback, event-level diagnostics, and physics-audit rules are used to reject physically invalid shortcut edits.

### 6.2. Relevance to Power-Engineering Fault Diagnosis

The proposed approach follows the broader practice of signal-based diagnosis in power engineering: faults are inferred from measured electrical signatures rather than direct internal observation. Here, terminal EMT waveforms act as the observable diagnostic signals, ADM/FDM/event indicators act as diagnostic descriptors, and the fault classification scheme acts as a decision-support layer. The extension to converter models or apparatus equivalents used in reliability studies is plausible but not validated in this feasibility study.

### 6.3. Diagnostic and Audit Contributions

The cases show complementary module roles. Event indicators localize the single-step InvSqrt and breaker faults that global metrics dilute, while ADM/FDM/GDM describe the distributed VARRL residual. Waveform features support source-level hypotheses, but retained breaker trajectories show that the first edit can worsen RMSE; compile, simulation, and waveform feedback, therefore, remain necessary.

The ten-candidate audit set addresses source admissibility separately. Three designed refactorings received Pass, six compilable rule violations received Reject, and one underspecified convention received Expert review. This author-constructed evidence does not provide independent classification rates, and the newly constructed candidates were compile-verified rather than executed in RTDS.

### 6.4. Behavioral-Oracle Insufficiency and Within-Case Evidence

Different source-level defects in the same coupled VARRL component can remain dormant or become observable depending on the applied R/L command event. Consequently, single-condition conformance testing can miss dormant implementation faults.

The R/L-step X1 result illustrates the constrained formulation of Section 2.1: although X1 produced a lower reference-based waveform loss than C0 (0.011782 versus 0.012893 in GDM), its audit decision was Reject, so X1 is excluded from the feasible repair set of Equation (3) despite its lower waveform discrepancy. A plausible explanation is fortuitous partial error cancellation between the snapshot inconsistency and the nominal model-to-reference mismatch; proximity to an opaque reference cannot distinguish such cancellation from genuine semantic correctness. Conversely, the L-only X2 result operationalizes the $h_{\mathrm{expert}}$ outcome: an activated source path whose behavioral effect remains within the matched REF–C0 discrepancy is routed to expert review rather than forced into a known class.

The challenge set further shows that compilation and admissibility are distinct: all six rule-violating candidates compiled, while the audit rejected them. Pass outcomes for the three refactorings show tolerance of alternative code organization, and the underspecified time-index candidate triggered Expert review rather than a forced decision.

### 6.5. Applicability Boundary Shown by the VARRL Case

The VARRL case shows a boundary of the current framework. The corrective actions are effective when the dominant error is associated with a single initialization or event-transition fault, as in InvSqrt and breaker. By contrast, VARRL involves coupled parameter filtering, rate limiting, Norton-equivalent conductance updates, and history-current re-latching. The current action set reduces the dominant transient error but does not guarantee exact waveform coincidence under such coupled discrete-time dynamics.

### 6.6. Limitations and Future Work

The evidence is limited to three project-derived cases and a within-case VARRL extension at one time step and command instant; the controlled variants are mechanism probes rather than independently observed faults. REF–C0 residuals are condition-specific comparators, not a stochastic solver-noise distribution, and subtle effects may still require expert review. The ten-candidate audit set covers each rule once across three structures, but has no independent human labels, and the newly constructed candidates were not executed in RTDS; it therefore cannot estimate classification or false-decision rates. The study also lacks multi-seed LLM runs, comparisons with monolithic agents, systematic failure logs, fully autonomous RTDS GUI execution, certified FSV metrics, and cross-platform validation.

Future work will focus on larger cross-component fault benchmarks, broader matched operating conditions and time steps, open-set fault detection, larger independently labeled challenge sets with dynamic RTDS execution of legitimate, inadmissible, and underspecified candidates, quantitative GUI-automation reliability tests, and cross-platform validation on other EMT simulation environments.

## 7. Conclusions

This paper studies waveform-feature-driven diagnosis and physics-constrained self-correction for representative EMT component implementation faults. Fault detection uses FSV-inspired waveform features and event-level anomaly detection; correction is performed through constrained source-level code updates under compile, behavioral-conformance, and applicability-aware audit feedback.

First-step gating removed the InvSqrt startup error of 0.5, and event-state restructuring removed the breaker error of 0.950469 at t=0.50075 s. The VARRL correction reduced node-voltage RMS relative differences to 0.098–0.099%.

Within the VARRL case, three controlled executable source variants demonstrated condition-dependent mechanism activation, including a weakly separated 0.018% RMS effect and a smaller activated-but-inseparable case assigned to expert review. Another variant reduced the reference-based waveform error but violated a discretization contract. Together with the three project-derived cases, these results support closed-set mechanism-level feasibility; statistical performance and cross-platform generality require broader evaluation.

All ten offline audit candidates were compiled in CBuilder; the audit returned Pass for three designed refactorings, Reject for six isolated rule violations, and Expert review for one underspecified convention. The results support mechanism-level feasibility and preliminary rule coverage, not an independent accuracy estimate or general rule sufficiency.

## Figures and Tables

**Table 1:** Physics-and-discretization audit rules.

Audit Rule	Violation Pattern	Default Outcome
**R1: State continuity**	Unconditional reset of dynamic states outside events or initialization	Reject
**R2: Discretization and snapshot consistency**	History variables overwritten before the current-step equation completes, or coupled terms using inconsistent same-step snapshots	Reject; unresolved if the time-index convention is unspecified
**R3: Event consistency**	Repeated or contradictory switching-state changes within one logical transition	Reject; unresolved for underspecified event semantics
**R4: Shortcut suppression**	Undeclared dead zone, clipping, or hard zeroing introduced only to reduce waveform error	Reject
**R5: Initialization closure**	First-step output inconsistent with the specified initialization semantics	Reject, with targeted repair guidance
**R6: Declared-constraint conformance**	Removal or bypass of a declared rate limiter, range limiter, or required filter	Reject

**Table 2:** Audit-rule applicability by component.

Audit Rule	InvSqrt	Breaker	VARRL
**R1: State continuity**	First-step latch	Event memory	History states
**R2: Snapshot consistency**	N/A	Rhis update	Norton and history snapshots
**R3: Event consistency**	N/A	Event logic	N/A
**R4: Shortcut suppression**	Proposed edits	Proposed edits	Proposed edits
**R5: Initialization closure**	First-step logic	N/A	N/A
**R6: Declared constraints**	N/A	N/A	Limits, filters, and rate limiter

*Note:* N/A: not applicable under the frozen component contract. Proposed edits: R4 is evaluated on proposed source-code changes rather than on the original implementation.

**Table 3:** Diagnostic evidence and actions for outcomes in H.

Diagnostic Evidence	Diagnostic Outcome	Action
Startup impulse or initialization bias	Initialization or latching fault: $h_{\mathrm{init}}$	Initialization gating, first-step bypass, or step counter
Localized switching-instant spike or zero-crossing chatter	Unstable event-transition logic: $h_{\mathrm{event}}$	Event restructuring, state smoothing, or history-memory insertion
Transient overshoot, drift, or residual mismatch	Inconsistent history-state update: $h_{\mathrm{history}}$	Memory re-latching, update reordering, or history correction
Discrepancy within the behavioral acceptance tolerance	No actionable fault: $h_{\mathrm{none}}$	Retain the candidate; no repair
Out-of-library or insufficient evidence	Expert review: $h_{\mathrm{expert}}$	Manual inspection; no forced correction

**Table 4:** Correction results for the three project cases.

Case	Fault Signature	Key Metrics (Before → After)	Status
InvSqrt	Startup single-sample pulse	GDM 0.023705→0; max error 0.5→0 at t=0	Corrected
Single-phase breaker	Switching-instant history-value error	Max error 0.950469 at t=0.50075 s →0; global GDM 0.000423→0	Corrected
VARRL	Distributed residual mismatch	RMS relative differences for the three phase-paired channels: 8.762%,18.522%,16.858%→0.098%,0.099%,0.099%; residual GDM 0.000979	Engineering-usable mitigation

**Table 5:** Global versus local-window FSV-inspired metrics for the pre-correction breaker waveform.

Scope	ADM	FDM	GDM	RMS rel. (%)
Global record	0.000092	0.000412	0.000423	0.042
Local window	0.001158	0.005180	0.005308	0.531

**Table 6:** Within-case activation, empirical separability, and interpretation of the VARRL source variants (200 ms transition window, phase-mean values).

Condition	Variant	C0–X_*i*_ RMS rel.	$R_{G}$	REF–X_*i*_/REF–C0	Interpretation
R-only	X1	∼$10^{-8}\%$	≈1	1.0000	Not activated
R-only	X2	0.01831%	$1.7\times 10^{6}$	1.0175	Activated; weak margin
R-only	X3	∼$10^{-8}\%$	≈1	1.0000	Not activated
L-only	X1	0.02749%	$2.4\times 10^{6}$	1.0987	Activated; modest margin
L-only	X2	0.000551%	$4.9\times 10^{4}$	0.9998	Activated; unresolved ($h_{\mathrm{expert}}$)
L-only	X3	0.89515%	$9.2\times 10^{7}$	6.4508	Clearly separable
R + L	X1	0.51655%	$4.6\times 10^{7}$	0.9138	Activated; lower REF error
R + L	X2	0.02015%	$1.8\times 10^{6}$	1.0126	Activated; weak margin
R + L	X3	12.82109%	$1.3\times 10^{9}$	10.0591	Clearly separable

**Table 7:** Deterministic audit outcomes for the inspectable VARRL implementations.

Implementation	Source Status	Audit Outcome	Basis
REF	Opaque black-box reference	Not source-audited	Reference source unavailable
C0	Retained corrected white-box	Pass	Consistent same-step snapshots; declared limiter retained
X1	Mixed inductance snapshots	Reject	*G* and $I_{\mathrm{hist}}$ use inconsistent *L* snapshots
X2	One-step parameter delay	Unresolved → expert review	Depends on intended time-index convention
X3	Rate-limiter bypass	Reject	Declared dL constraint removed

**Table 8:** Offline cross-component audit challenge set: construction roles, compile feasibility, and deterministic audit outcomes.

Candidate	Component	Construction Role	Target Rule	Compile/Audit
VAR_EQ	VARRL	Designed refactoring	—	Pass/Pass
BRK_EQ	Breaker	Designed refactoring	—	Pass/Pass
INV_EQ	InvSqrt	Designed refactoring	—	Pass/Pass
VAR_RST	VARRL	State clearing	R1	Pass/Reject
VAR_F1_MIXL (X1)	VARRL	Mixed snapshots	R2	Pass/Reject
BRK_CHAT	Breaker	Contradictory event commits	R3	Pass/Reject
VAR_DZ	VARRL	Undeclared dead zone	R4	Pass/Reject
INV_NOINIT	InvSqrt	Missing initialization closure	R5	Pass/Reject
VAR_F3_NORATE (X3)	VARRL	Limiter bypass	R6	Pass/Reject
VAR_F2_LAG1 (X2)	VARRL	Underspecified time index	R2	Pass/Expert review

## Data Availability

The data that support the findings of this study are available from the corresponding author upon reasonable request. Some platform-specific project files, source-code implementation details, and benchmark model files are not publicly available due to institutional and project restrictions. The waveform comparison script, exported CSV-based metric summaries, offline sensitivity-analysis summaries, final offline audit matrix, compile checklist, frozen rule/contract summaries, and anonymized trajectory summaries can be made available from the corresponding author upon reasonable request. Raw trajectory logs are not released directly because they contain local file paths and sensitive runtime metadata.
